# Dynamic physiological response of tef to contrasting water availabilities

**DOI:** 10.3389/fpls.2024.1406173

**Published:** 2024-07-09

**Authors:** Muluken Demelie Alemu, Vered Barak, Itamar Shenhar, Dor Batat, Yehoshua Saranga

**Affiliations:** ^1^ The Robert H. Smith Faculty of Agriculture, Food and Environment, The Hebrew University of Jerusalem, Rehovot, Israel; ^2^ Crop Research, Ethiopian Institute of Agricultural Research (EIAR), Addis Ababa, Ethiopia

**Keywords:** critical drought point, drought adaptation, drought recovery, drought stress, productivity, transpiration, tef/teff, water-use efficiency

## Abstract

Global climate change is leading to increased frequency of extreme climatic events, higher temperatures and water scarcity. Tef (*Eragrostis tef* (Zucc.) Trotter) is an underutilized C4 cereal crop that harbors a rich gene pool for stress resilience and nutritional quality. Despite gaining increasing attention as an “opportunity” crop, physiological responses and adaptive mechanisms of tef to drought stress have not been sufficiently investigated. This study was aimed to characterize the dynamic physiological responses of tef to drought. Six selected tef genotypes were subjected to high-throughput whole-plant functional phenotyping to assess multiple physiological responses to contrasting water regimes. Drought stress led to a substantial reduction in total, shoot and root dry weights, by 59%, 62% and 44%, respectively (averaged across genotypes), and an increase of 50% in the root-to-shoot ratio, relative to control treatment. Drought treatment induced also significant reductions in stomatal conductance, transpiration, osmotic potential and water-use efficiency, increased chlorophyll content and delayed heading. Tef genotypes exhibited diverse water-use strategies under drought: water-conserving (isohydric) or non-conserving (anisohydric), or an intermediate strategy, as well as variation in drought-recovery rate. Genotype RTC-290b exhibited outstanding multifaceted drought-adaptive performance, including high water-use efficiency coupled with high productivity under drought and control treatments, high chlorophyll and transpiration under drought, and faster drought recovery rate. This study provides a first insight into the dynamic functional physiological responses of tef to water deficiency and the variation between genotypes in drought-adaptive strategies. These results may serve as a baseline for further studies and for the development of drought-resistant tef varieties.

## 1 Introduction

Climate change is leading to increased frequency of extreme climatic events, higher temperatures and water scarcity, resulting in a global reduction in crop production (Kim et al., 2020; Joshi et al., 2021; Correia et al., 2022). Underutilized (also known as orphan) crop species, which are not widely cultivated, are highly relevant to food security for millions of people, and yet they have not been sufficiently studied or improved (Tadele, 2019; Allaby, 2021; Milla and Osborne, 2021). Underutilized crops harbor a rich gene pool for improvement (Chapman et al., 2022) and resilience to various stresses (VanBuren et al., 2020), thus offering the potential to improve food and nutrition security (Mabhaudhi et al., 2019; Siddique et al., 2021).

Tef [*Eragrostis tef* (Zucc.) Trotter] is one such underutilized cereal crop (Assefa et al., 2011; Tadele, 2019; VanBuren et al., 2020). Ethiopia serves as the center of origin and diversity for tef (Vavilov, 1951), where it plays a crucial role as a staple crop for food and feed (D’Andrea, 2008; Assefa et al., 2015, 2017; Chanyalew et al., 2019), as well as a source of income for smallholder farmers (Paff and Asseng, 2018). Tef grains are gluten-free and rich in minerals, essential amino acids, fiber and vitamins (Shumoy et al., 2018; Abewa et al., 2019; Tietel et al., 2020; Ligaba-Osena et al., 2021; Villanueva et al., 2022), prompting its global recognition as a nutritious “superfood”. Tef has a C_4_ photosynthetic apparatus that is well-adapted to high temperatures and radiation, and it exhibits resilience to various abiotic and biotic stresses (Assefa et al., 2011; Tadele, 2019; Girma et al., 2020; VanBuren et al., 2020).

Under drought, plants deviate from optimal growth conditions, limiting their capacity to realize their full potential at various growth stages (Moshelion, 2020; Joshi et al., 2021). Plant responses to drought stress are complex, involving a range of physiological reactions from perception to the implementation of stress-resistance strategies at the cellular to whole-plant levels (Takahashi et al., 2020). Physiological traits exhibit high plasticity, with changes ranging from hourly to seasonal in response to environmental conditions (Dalal et al., 2017; Moshelion, 2020; Li et al., 2021). Plant responses to drought can be quantified through morphological, biochemical and physiological traits (Wu et al., 2021). These responses encompass alterations in physiological processes, root growth and architecture, phenology, growth and development, ultimately leading to reduced productivity (Moshelion et al., 2015; Gupta et al., 2020). Vapor pressure deficit (VPD) is the key driving force for water movement from the soil to the plant, affecting stomatal conductance along with soil water content (Moshelion et al., 2015; Appiah et al., 2023).

Water-use efficiency (WUE), defined as the amount of carbon gain (carbon fixation) relative to water use (transpiration), is a key target for crop improvement (Leakey et al., 2019). WUE represents a vital physiological measure of how plants use water effectively to produce biomass or yield and mitigate the effects of drought stress (Gupta et al., 2020). Crop yield depends on the interplay between transpiration rate (TR) and WUE, with WUE tending to decrease with increasing TR and vice versa. However, achieving high yield and WUE in plant breeding programs has proven challenging due to gaps in our understanding of the interactions between physiological and yield traits (Sun, 2023).

Plant responses to drought exhibit reversibility (recovery), the extent of which varies with the duration and severity of the stress, and the plant’s developmental stage and genetic makeup (Wang et al., 2022). During the recovery phase, following water resumption, physiological functions swiftly revert to their normal levels (Chen et al., 2016; Appiah et al., 2023). Drought recovery is recognized as crucial for plant adaptation to drought conditions, and a more important phenomenon than previously acknowledged (Chen et al., 2016).

While plants exhibit remarkable phenotypic and physiological changes in response to environmental conditions (Moshelion, 2020), the development of drought- resilient varieties remains limited, primarily due to the absence of real-time functional/physiological phenotyping (Vera-Repullo et al., 2015; Li et al., 2021). The comprehensive phenotyping of whole-plant function and physiology is challenging when relying on manual measurements (Vera-Repullo et al., 2015). High-throughput phenotyping tools offer non-destructive and non-invasive methods, facilitating accurate and rapid whole-plant multiphase functional phenotyping under various treatments (Joshi et al., 2021; Pandey et al., 2021). These tools are invaluable for detecting the physiological dynamics of plant responses to drought on a large scale, and contribute to a comprehensive understanding of physiological traits (Dalal et al., 2020; Kim et al., 2020).

Tef exhibits diverse responses to drought stress, e.g., changes in photosynthetic and transpiration rates, osmotic adjustment, leaf water potential, root development, leaf rolling, electrolyte leakage, and protein and metabolite contents (Ayele et al., 2001; Balsamo et al., 2006; Degu et al., 2008; Mengistu, 2009; Ginbot and Farrant, 2011; Kamies et al., 2017; Girija et al., 2022). Tef is resilient to drought and exhibits variation in recovery from stress, although severe drought stress can lead to irreversible damage (Ginbot and Farrant, 2011; Kamies et al., 2017). Nevertheless, the physiological phenotyping of tef in response to drought stress is currently inadequate (Girija et al., 2022); in particular, whole-plant dynamic drought-adaptive reactions have not been sufficiently characterized.

In our previous study, we documented phenological, morpho-physiological, lodging, and productivity traits of a wide tef germplasm collection under contrasting water regimes, and identified their underlying genomic loci (Alemu et al., 2024). Here, we focus on six selected tef genotypes and characterize their dynamic physiological responses to contrasting water regimes. The outcomes of this study are expected to shed light on tef’s responses to water availability, toward the development of drought-resilient varieties.

## 2 Materials and methods

### 2.1 Functional phenotyping system

The study was conducted at the Israeli Center of Research Excellence (iCORE) (http://departments.agri.huji.ac.il/plantscience/icore.Phpon) at the Robert H. Smith Faculty of Agriculture, Food and Environment, The Hebrew University of Jerusalem, Rehovot, Israel. iCORE is a functional physiological phenotyping facility, consisting of the Plantarray 3.0 system (Plant-DiTech, Israel) in a semi-temperature-controlled greenhouse (Dalal et al., 2020; Pandey et al., 2021). The system consists of highly sensitive gravimetric lysimeters, soil and atmospheric probes, data acquisition units and a precise irrigation controller. The system enables continuous assessment of plant–water relations and soil and atmosphere parameters throughout the plant’s entire growth season.

Plants were grown in pots, with each pot connected to its own irrigation controller and data-acquisition unit. Data were recorded automatically every 3 min and saved in a server. Atmospheric variables—air temperature, relative humidity (RH), VPD, and photosynthetically active radiation (PAR)—were recorded in the Plantarray system.

### 2.2 Plant materials and experimental layout

Tef genotypes were selected from our tef diversity panel (TDP-300) based on a previous study (Alemu et al., 2024). Genotypes with similar, medium phenology (heading time), but diverse productivity were selected based on their performances in a replicated field experiment conducted in 2021 (**Supplementary Table S1**).

A two-way factorial experiment was conducted with six tef genotypes x two treatments (control and drought) and six replicates, altogether 6 x 2 x 6 = 72 pots (experimental units). A random block design was used to ensure uniform exposure to the environment and minimize variations. Load-cell units were calibrated under constant load weights (1 kg and 5 kg) using the Plantarray software. Plastic drainage containers were positioned on the Plantarray lysimeters before transferring the pots into the system, to ensure the accuracy of lysimeter weighing and minimize noise in the system. The initial total pot weight, comprising all components, was incorporated into the Plantarray system before the experiment was initiated. This included weight of the pot, drainage container, pot soil, water in the drainage container, stick and trilling rope, and seedlings’ initial fresh weight. The later was determined through destructive harvest on the day of transplanting (28 days after sowing), averaging the weights of nine seedlings per genotype.

Seedling trays and growing pots were sterilized and thoroughly washed before being filled with the growing medium. Seedling trays (10 ml cone) were filled with growing medium Matza Gan (Shaham, Givat-Ada, Israel). Tef seeds were sown on 22 May 2022 in seedling trays (~4 seeds cone^-1^), watered manually once a day, and maintained in the iCORE greenhouse for 28 days. Thinning was conducted after 1 week to 1 seedling cone^-1^.

**Figure 1:**
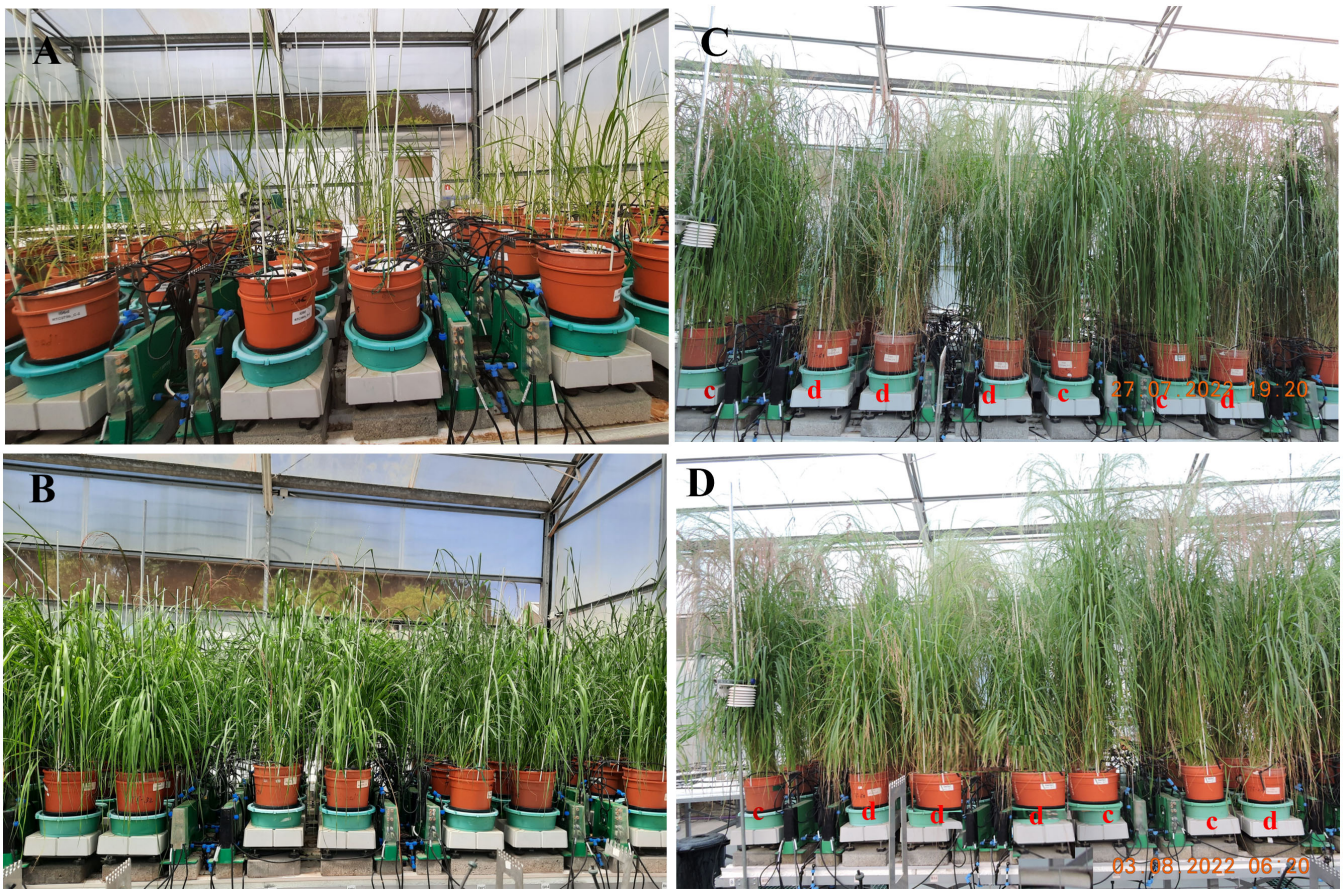
Partial view of the tef functional phenotyping experiment consisting of 72 measuring units loaded on the Plantarray system. **(A)** day 1 - seedling transplanted into the pots; **(B)**, day 13 - plants at end of pretreatment phase; **(C)** day 37 - control (c) and drought (d) treated plants at the end of differential treatment phase; **(D)** day 43 - control (c) and drought (d) treated plants at the end of recovery phase.

Plastic pots (4 l) were filled with silica sand 20/30 mesh (Negev Industrial Minerals Ltd., Israel) growing medium and washed with water prior to transplanting to eliminate pore spaces. To minimize evaporation, the soil surface was covered with a white polyethylene-vinyl acetate with three equally spaced planting holes. Transplanting was used to establish a single plant in each planting hole. Three seedlings were transplanted per pot (12 pots per genotype) and each pot was placed in a plastic drainage container at its designated position on a Plantarray lysimeter and connected to four outlet drippers to ensure uniform soil moisture (**Figure 1A**). Pre-experimental observation of tef daily transpiration (DT) was used as a reference to determine the initial daily irrigation amount. Irrigation was applied four times during the night (2000 h–0300 h) to minimize “noise” during data measurements. The pot soil’s volumetric water content (Cθ) was about 1200 ml, with 80 ml of water remaining in the drainage containers, providing extra water to the control plants beyond the pot soil ‘s capacity.

The irrigation program was designed to apply differential irrigation treatments (control and drought stress) based on each pot’s transpiration. In the control group, each pot was irrigated at a rate of ~130% relative to its transpiration on the previous day. Water application to the drought-treated genotypes consisted of three phases: pretreatment (13 days, identical to control) (**Figure 1B**), differential irrigation (24 days) (**Figure 1C**), and recovery (6 days) (**Figure 1D**). To avoid rapid water depletion and to mimic the development of soil water deficits in the field, water application was gradually reduced by ~60 ml of each pot’s previous day’s transpiration for 11 days (day 14 to 24). Irrigation remained at this level for 10 days (day 25 to 34), and was then further reduced over 3 days (day 35 to 37) to provide 50% of the previous day’s transpiration. Finally, on day 38, at the onset of the recovery period, full irrigation was resumed until the final harvest on 3 August 2022 (43 days after transplanting).

Environmental conditions in the greenhouse throughout the experimental season were on average (min/max): temperature 22.7/31.1°C, RH 45.5/80.5% and VPD 0.62/2.41 kPa, with an average maximum PAR of 1318 μmol m^-2^ s^-1^ (**Supplementary Figure S1**). Commercial fertilizers were applied through the drip irrigation system (fertigation), providing 67, 10 and 42 ppm N, P and K, respectively, and micronutrients. No pesticide application was required during the experimental season.

### 2.3 Whole-plant functional phenotyping

High-throughput phenotyping platform was employed to continuously monitor whole-plant functional physiological traits related to plant–water–soil–atmosphere kinetics. TR, DT, normalized transpiration (E), canopy stomatal conductance (G_sc_) and calculated fresh weight (CFW) were either directly recorded or estimated by the Plantarray system. Real-time data inspection and analysis were carried out using Soil-Plant-Atmosphere-Continuum (SPAC) Analytics web-based software (Plant-DiTech, Israel) (Dalal et al., 2017; Halperin et al., 2017; Gosa et al., 2022).

A conventional phenotyping approach was used to assess phenology, physiology and productivity traits. Days from planting to heading were recorded based on visual observations. Leaf osmotic potential (OP), measured as previously described by Alemu et al. (2024), and chlorophyll content (Chl), measured using a SPAD 502Plus chlorophyll meter (Konica Minolta, Japan), were recorded twice: at the end of the differential irrigation treatment and at the end of the recovery/experimental period. Productivity traits, including shoot, root and total dry weights (SDW, RDW and TDW, respectively), were measured following destructive harvest. Shoot and root biomass were separated, roots were thoroughly washed, and both parts were oven-dried (60°C, 74 h) and weighed.

### 2.4 Data processing and statistical analysis

JMP® version 16 Pro statistical package (SAS Institute, Cary, NC, USA) was used for ANOVA to test the effects of genotype, treatment and their interactions, as well as for correlation analyses.

Piecewise curve fitting was conducted using the SPAC Analytics software to estimate the relationships between midday (1200–1400 h) TR and Cθ for each of the drought-treated pots, enabling a calculation of critical drought point (θ_crit_) and the slope of the TR reduction at Cθ < θ_crit_.

To assess the drought-recovery rate, we divided DT and CFW by their value on the first recovery day to calculate their relative values (RDT and RCFW, respectively). These relative values were correlated vs. recovery day using a linear regression, to determine their slopes (recovery rate). Differences between slopes of the tested genotypes were examined using GraphPad prism, Version 10.0.1 (GraphPad Software, Boston, MA, USA).

Relative total dry weight (RTDW), calculated as TDW under drought relative to TDW under control conditions, was used to estimate the effect of drought on productivity. WUE was calculated for the pretreatment phase based on plant fresh weight as: WUE_fw_ = ΔCFW_1-13_/ΣTR_1-13_, where ΔCFW_1-13_ is the difference between the calculated CFW on days 1 and 13 and ΣTR_1-13_ is the cumulative transpiration for days 1 to 13. For the entire season, dry weight-based WUE was calculated as WUE_dw_ = TDW_43_/ΣTR_1-43_, where TDW_43_ is TDW on day 43 and ΣTR_1-43_ is the cumulative transpiration for the entire growing period (Leakey et al., 2019).

## 3 Results

### 3.1 Seasonal dynamics of tef responses to contrasting water availabilities

**Figure 2:**
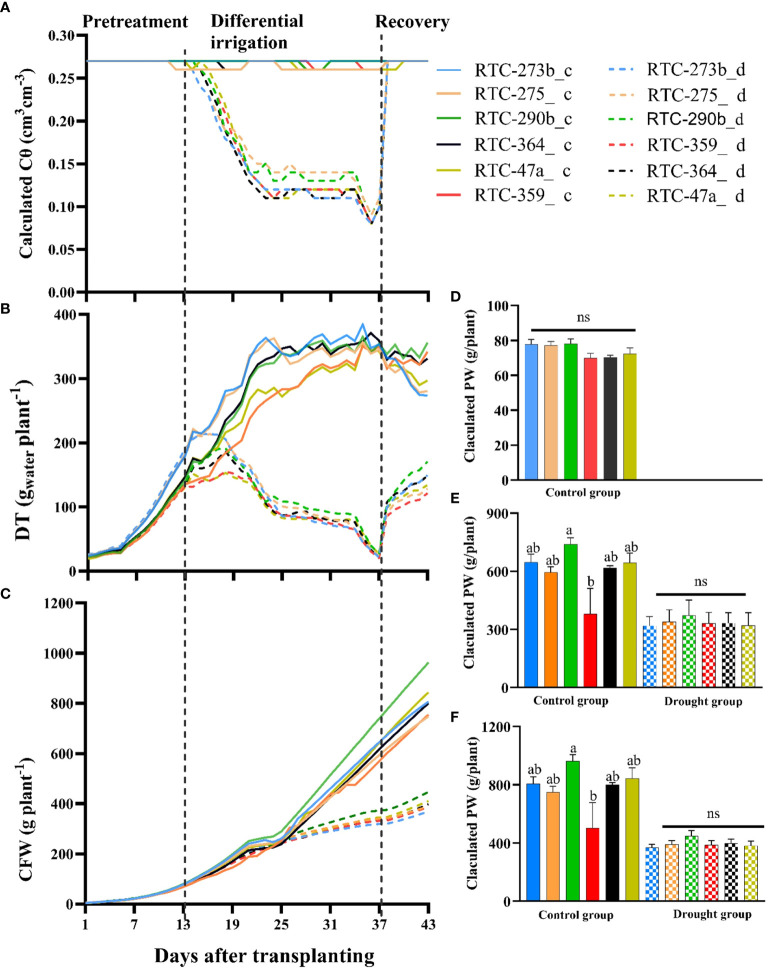
Tef response to water availability assessed continuously for 43 days under control (c) and drought (d) treatments: **(A)** calculated soil volumetric water content (Cθ); **(B)** daily transpiration rate; **(C)** calculated fresh weight (CFW); **(D)** CFW at the end of pretreatment phase (day 13); **(E)** CFW at the end of differential irrigation phase (day 37); **(F)** CFW at the end of recovery phase (day 43). Different letters indicate significant differences between genotypes (*p* < 0.05); ns, non-significant.

Cθ in the control group remained at 26–27% (apparently soil field capacity) throughout the entire experiment (**Figure 2A**). In the drought-treated group, Cθ was similar to the control during pretreatment phase (days 1–13), decreased gradually from day 14 to 24, stabilized at 11–13% on days 25–34, further decreased on days 35–37 reflecting the extreme drought applied, and then increased back to field capacity at the onset of the recovery phase.

During pretreatment phase, DT exhibited a gradual increase with highly significant variation (*p <*0.01) between genotypes (**Figure 2B**, **Supplementary Table S2**). Then, during differential irrigation, it continued to increase in the control group until about day 25 when it leveled off (**Figure 2B**). At the same time, the drought-treated group exhibited a gradual reduction in DT, even when Cθ was rather stable (days 25–34). Significant variations were observed in DT between treatments (all days) and genotypes (most days) with no genotype by environment interactions (**Supplementary Table S2**). Under control conditions, genotypes RTC-273b and RTC-359 consistently showed the highest and lowest DT, respectively, whereas under the drought treatment, RTC-290b and RTC-273b usually presented the highest and lowest DT, respectively. However, under extreme drought, toward the end of the differential treatments, only minor variation was observed between genotypes.

Following 24 days of differential water application, all plants were subjected to full irrigation (**Figure 2A**), thus allowing the drought-treated plants to rapidly absorb water and increase DT (**Figure 2B**, **Supplementary Table S2**). During the 6-day recovery period, all genotypes in the drought group exhibited a rapid increase in DT, while genotypes in the control group displayed either stable or reduced DT as compared to the differential irrigation phase. Highly significant variations were observed in DT between treatments (**Supplementary Table S2**). Consistent variations were observed between the genotypes; RTC-290b showed the highest DT under both treatments, whereas the lowest values under control conditions were recorded for RTC-273b and RTC-275, and under drought conditions for RTC-359.

CFW of the tef genotypes was recorded across the entire experiment, using non-destructive measurements (**Figure 2C**). During the pretreatment, there was a substantial increase in CFW with significant differences between genotypes observed during most days (**Figure 2D**, **Supplementary Table S3**). During the differential irrigation, the drought-treated group exhibited slower biomass accumulation compared to the control group. Variations in CFW between treatments became highly significant at the middle of the differential irrigation phase, while differences between genotypes and genotype by environment interactions became consistently significant at the last 4 days (**Supplementary Table S3**). At the end of this phase, significant variation was noted in CFW within the control group, whereas within the drought-treated group, no significant variation was evident (**Figure 2E**). During the recovery phase, CFW was significantly affected by genotypes, treatments and their interactions (**Supplementary Table S3**). Furthermore, at the end of the recovery period, significant variation was observed in CFW between genotypes in the control group, but not between the drought-treated genotypes (**Figure 2F**). RTC-290b consistently demonstrated the best performance in terms of CFW under both control (significant) and drought (non-significant) treatments throughout all periods, while RTC-359 and RTC-273b exhibited the lowest performance under control (significant) and drought (non-significant) treatments, respectively (**Figures 2D–F**).

### 3.2 Diurnal dynamics of tef responses to contrasting water availabilities

**Figure 3:**
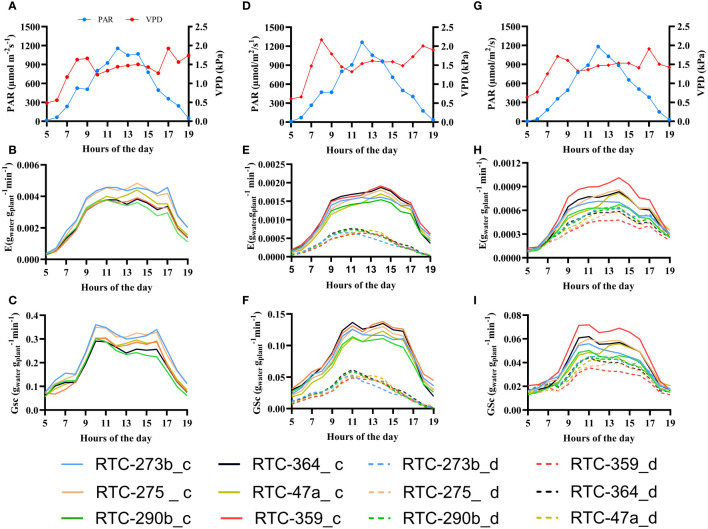
Daily patterns of vapor pressure deficit (VPD, red line), photosynthetically active radiation (PAR, blue line), canopy stomatal conductance (G_sc_) and normalized transpiration rate **(E)** in the three selected days under control (c) and drought (d) treatments: **(A–C)** pretreatment (day 10); **(D–F)** differential irrigation (day 28); **(G–I)** recovery (day 40).

The diurnal patterns of G_sc_ and E, for selected days of each experimental phase, are presented in **Figure 3** alongside PAR and VPD. Tef genotypes exhibited variations in G_sc_ and E in response to the diurnal pattern of VPD and PAR, with a major effect of Cθ. Both G_sc_ and E displayed, in most cases, a rapid increase during the morning (0500–1000 h), followed by stable values throughout midday (1000–1600 h) and a decrease in the late afternoon (1600–1900 h), following the patterns of PAR. A clear deviation from this trend was observed in the drought-treated plants which displayed their maximum G_sc_ and E at about 1100 h and a continuous decrease thereafter until the evening.

### 3.3 Dynamic responses to drought reflect variation in critical drought point and recovery

**Figure 4:**
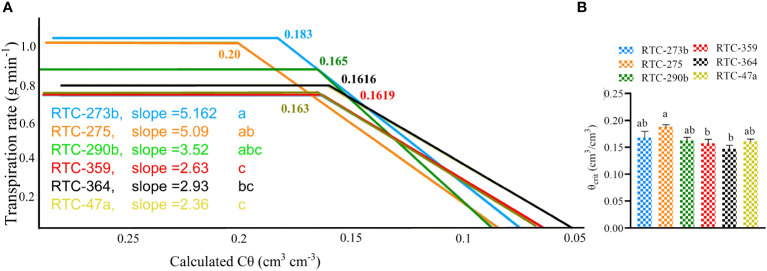
The relationships between midday transpiration rate (TR) and calculated soil volumetric water content (Cθ): **(A)** Piecewise curve fits in which the horizontal parts indicate the maximum TR, breaking points indicate critical drought point (θ_crit_) and slopes indicate the rate of TR reduction; **(B)** Comparison between θ_crit_ values of the various genotypes. Probabilities of differences between θ_crit_ values and slopes are indicated. Different letters (a,b,c) indicate significant differences between genotypes (*p* < 0.05).

The relationships between mid-day TR and Cθ under the drought treatment, revealed significant variation between genotypes in their responses to water availability. Two genotypes, RTC-273b and RTC-275, exhibited the highest TR (>1 g min^-1^) under high to moderate Cθ, while RTC-359 and RTC-47a exhibited the lowest values (~0.74 g min^-1^) (**Figure 4A**). Tef genotypes reflected significant variation in θ_crit_ (*p* = 0.001), with RTC-275 exhibited the highest level (0.20 cm^3^/cm^3^), significantly different from two genotypes, whereas RTC-273b exhibited an intermediate level (0.18 cm^3^/cm^3^), not differing from the highest or lowest genotypes (0.162 cm^3^/cm^3^) (**Figure 4B**). The high-transpiring genotypes (RTC-275, RTC-273b) exhibited high values of θ_crit_, whereas the medium-transpiring (RTC-290b) and low-transpiring (RTC-47a, RTC-359, RTC-364) genotypes exhibited lower θ_crit_. As Cθ was further reduced below θ_crit_, TR declined rapidly at rates (slopes) which differed significantly (*p* < 0.0002) between genotypes. RTC-273b and RTC-275 exhibited the fastest reduction rates, whereas RTC-364, RTC-359 and RTC-47a showed slowest rates, and RTC-290b reflected an intermediate reduction rate that did not differ from any of the other genotypes.

**Figure 5:**
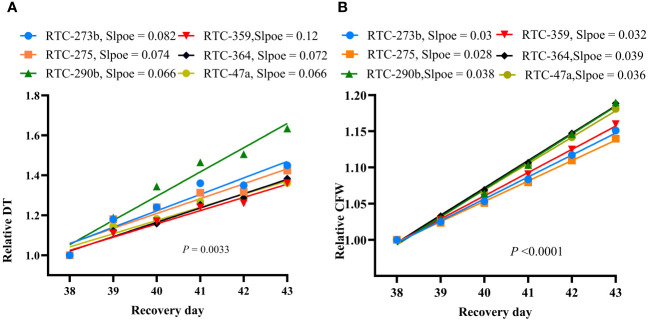
Drought-recovery rate of tef upon resumption of full irrigation after 24 days of drought stress: **(A)** Linear regression between relative daily transpiration (RTD) and day of recovery period; **(B)** Linear regression between relative calculated fresh weight (RCFW) and day of recovery period. Probabilities of differences between slopes are indicated in each graph.

Following the resumption of full irrigation, all genotypes exhibited remarkable recovery from drought stress, as reflected by increasing DT and CFW (**Figure 2**, **Supplementary Tables S2**, **S3**). The slopes of RDT and RCFW during the recovery phase displayed significant differences (*p* = 0.003 and *p* < 0.0001, respectively) between genotypes (**Figure 5**). RTC-290b exhibited the greatest RDT slope (recovery rate), significantly different from all other genotypes (**Figure 5A**), whereas for RCFW, RTC-290b, RTC-364 and RTC-47 exhibited significantly greater slopes than the other genotypes (**Figure 5B**).

### 3.4 Effects of drought on phenology and single-point physiological traits

Tef phenology (days from planting to heading) was significantly affected by water availability and genotype (**Table 1**). Drought stress imposed, on average, a ~6-day delay in heading time; however, genotypic ranking across treatments was similar. Heading time under both treatments was earliest in genotypes RTC- 273b and RTC- 275, followed by RTC-364 and RTC-47a, and ending with RTC-290b and RTC-359.

**Table 1:** ANOVA for the effects of water availability and genotype on days from planting to heading (DPH), chlorophyll contents (Chl) and osmotic potential (OP) at the end of the differential irrigation phase (ChlD and OPD, respectively), and after recovery (ChlR and OPR, respectively).

Source	DF	DPH (day)	OPD (MPa)	OPR (MPa)	ChlD (SPAD value)	ChlR (SPAD value)
Treatment Genotype Treat*Geno Block	1 5 5 5	129.5^***^ 15.9^***^ 1.2 1.0	109.1^***^ 5.2^**^ 1.8 3.1	0.04 7.2^***^ 0.7 3.4^*^	9.8^**^ 3.8^**^ 2.7^*^ 0.4	0.4 2.9^*^ 1.3 0.7
Treatment effect						
Control Drought		57.4b 63.0a	-1.18a -1.49b	-1.13a -1.14a	34.7b 36.5a	34.9a 35.3a
Genotype effect						
Control						
RTC-273b RTC-275 RTC-290b RTC-359 RTC-364 RTC-47a		55.0c 55.0c 59.5ab 59.8a 57.3b 57.7ab	-1.05a -1.13a -1.17a -1.23a -1.28a -1.22a	-1.02a -1.05ab -1.15ab -1.22b -1.20ab -1.19ab	35.4ab 33.4ab 32.8b 35.2ab 37.2a 34.4ab	34.0a 33.3a 33.5a 35.0a 37.5a 35.9a
Drought						
RTC-273b RTC-275 RTC-290b RTC-359 RTC-364 RTC-47a		61.3bc 59.8c 65.0ab 67.3a 63.5abc 61.0bc	-1.46ab -1.32a -1.51b -1.63b -1.53b -1.47ab	-1.06a -1.08a -1.08a -1.26b -1.18ab -1.14ab	35.1b 34.3b 36.2ab 37.2ab 36.8ab 39.7a	34.5ab 32.4b 36.7ab 34.1ab 35.7ab 38.7a

DF, degrees of freedom; *, ** and, *** indicate significant F ratio at p < 0.05, 0.01 and 0.001, respectively. Different letters (a, b) denote significant differences between treatments (t-test) and genotypes (Tukey HSD test) at p < 0.05.

The impact of drought stress was also evident at the end of the differential irrigation period as a 0.31 MPa lower OP under drought stress compared to the control (**Table 1**). While under control conditions, no significant differences were found between genotypes, the drought-treated group reflected significant variation, with three genotypes (RTC-290b, RTC-359 and RTC-364) showing the lowest values. After recovery for 6 days, all genotypes showed increased OP, to a level similar to the control, with RTC-359 showing the lowest values under both treatments. Drought stress induced an increased Chl content, however responses of various genotypes were inconsistent (**Table 1**).

### 3.5 Productivity and WUE

ANOVA revealed significant differences between treatments and genotypes for most productivity variables, and non-significant treatment-by-genotype interactions (**Table 2**). Drought stress led to a substantial reduction in TDW, SDW and RDW, by 59%, 62% and 44%, respectively (average across genotypes) (**Table 2**, **Supplementary Figure S2**), and an increase of 50% in the RDW-to-SDW ratio, relative to control conditions. Notably, RTC-290b displayed the highest TDW and SDW, while RTC-47a exhibited the lowest values under both control (not significant) and drought treatments. On the other hand, no significant differences were detected between genotypes in RDW and RDW-to-SDW ratio under either treatment. In addition, the analysis of RTDW revealed non-significant differences between genotypes, with RTC-290b and RTC-275 exhibiting the highest values.

**Table 2:** ANOVA for the effects of water availability and genotype on total dry weight (TDW), shoot dry weight (SDW), root dry weight (RDW), root-to-shoot dry weight ratio (RDW/SDW), relative total dry weight (RTDW), and fresh weight- and dry weight-based WUE (WUE_fw_ and WUE_dw_, respectively).

Source	DF	TDW (g plant^-1^)	SDW (g plant^-1^)	RDW (g plant^-1^)	RDW/ SDW	RTDW	WUE_fw_ (g kg^-1^)	WUE_dw_ (g kg^-1^)
Treatment Genotype Treat*Geno Block	1 5 5 5	387.70^***^ 2.94^*^ 0.37 1.52	440.11^***^ 3.63** 0.24 1.16	90.84^***^ 1.82 2.15 2.50	29.12^***^ 2.40* 1.25 3.43*	0.48 0.58	3468.90* 1064.20	5.08* 8.40*** 0.61 2.20
Treatment effect								
Control Drought		237.00a 97.00b	199.48a 75.99b	37.18a 20.97b	0.18b 0.27a			7.9a 7.6b
Genotype effect								
Control								
RTC-273b RTC-275 RTC-290b RTC-359 RTC-364 RTC-47a		243.6a 233.9a 259.2a 228.8a 251.8a 206.1a	206.7a 202.7a 220.0a 187.0a 208.0a 174.0a	36.9a 29.6a 39.0a 41.7a 43.8a 32.1a	0.13a 0.18a 0.19a 0.21a 0.19a 0.18a		83.8b 83.3b 105.0a 101.2ab 93.7ab 98.0ab	7.7ab 7.3ab 8.6a 8.5ab 8.3ab 7.1b
Drought								
RTC-273b RTC-275 RTC-290b RTC-359 RTC-364 RTC-47a		94.3ab 99.2ab 113.1a 93.7ab 100.1ab 80.3b	76. 7ab 77.3ab 90.4a 69.8ab 80.6ab 59.5b	17.6a 22.4a 22.7a 22.8a 19.6a 20.8a	0.23a 0.23a 0.26a 0.28a 0.24a 0.35a	0.39a 0.43a 0.44a 0.41a 0.40a 0.39a		6.9b 7.4ab 8.2a 7.8ab 8.2a 6.9b

DF, degrees of freedom; *, ** and *** indicate significant F ratio at p < 0.05, 0.01 and 0.001, respectively. Different letters (a, b) denote significant differences between treatments (t-test) and genotypes (Tukey HSD test) at p < 0.05.

Significant differences were found between genotypes for the pretreatment WUE_fw_, whereas for WUE_dw_, differences between both genotypes and treatments were significant (**Table 2**). A rather small, albeit significant reduction in WUE_dw_ was recorded under drought relative to control conditions. RTC-290b exhibited the highest WUE in all cases under both treatments, the lowest WUE values were noted for RTC-47a under control conditions, and for RTC-273b and RTC-47a under drought, whereas all other genotypes exhibited intermediate levels.

## 4 Discussion

Drought is one of the most severe environmental stresses affecting pivotal physiological, developmental and metabolic processes in plants, ultimately reducing growth and productivity (Brodersen et al., 2019; Kerchev and Van Breusegem, 2022). Tef is a self-pollinated, annual C_4_ cereal crop that is resilient to various environmental and biotic stresses (Assefa et al., 2011; Kamies et al., 2017; Girija et al., 2022; Alemu et al., 2024), but its stress-adaptive mechanisms have not been sufficiently studied. In response to drought stress, tef exhibits changes in transpiration, Chl, photosynthesis, electrolyte leakage, ultrastructure, protein content and metabolites (Ginbot and Farrant, 2011; Kamies et al., 2017; Girija et al., 2022). In the current study, we utilized a high-throughput functional phenotyping system to characterize the dynamic physiological responses underlying drought adaptation and productivity in tef.

### 4.1 Transpiration dynamics of tef genotypes reflect response to water availability

Transpiration under drought stress can differentiate resistant from susceptible genotypes (Bacher et al., 2022). Plants that exhibit high transpiration under optimal water supply, and a moderate decrease under drought, combined with stress resilience, can secure both high and stable productivity across a range of water availabilities (Appiah et al., 2023). During drought stress, plants shift into a survival mode at the expense of their productivity, thus reducing transpiration and carbon fixation as compared to controls (Moshelion, 2020). Tef genotypes exhibited increasing DT during the pretreatment phase, which has rapidly decreased upon exposure to drought stress (**Figure 2**, **Supplementary Table S2**). During the differential irrigation phase, plants exhibited variations in DT between treatments and genotypes, suggesting G_sc_-regulated changes in gas exchange. During the recovery phase, the drought-treated plants responded immediately to re-watering by increasing their DT and CFW accumulation (**Figure 2**). During all three phases and under both treatments, tef genotypes manifested considerable differences in DT, E and G_sc_, presumably reflecting genetic variation (**Figures 2**, **3**, **Supplementary Table S2**), which ultimately led to differences in productivity and WUE (**Table 2**). Compared to the other genotypes, RTC-290b exhibited moderate DT under control and high DT under drought (**Figure 2**), as well as rapid recovery (**Figure 5**), indicating its resilience to water stress.

The interactions between VPD, PAR, G_sc_ and E are crucial to maintaining physiological processes and optimizing plant water use and productivity under different water regimes (Gosa et al., 2022). VPD plays a vital role in water transport and regulation of stomatal conductance (Appiah et al., 2023). Drought-treated plants displayed a distinctly different diurnal pattern in G_sc_ and E compared to the control group (**Figure 3**), suggesting different regulation and water-use strategy. Upon resumption of full irrigation, G_sc_ and E showed similar diurnal patterns under both treatments, with somewhat lower values in the drought-treated group, reflecting rapid recovery from stress.

Osmotic adjustment (i.e., reducing OP) is a common plant response to drought stress, enabling the maintenance of water absorption, turgor pressure and structural integrity (Turner, 2017; Condorelli et al., 2022). Similar to our previous study (Alemu et al., 2024), tef plants exhibited a significant reduction of OP in response to drought (**Table 1**). It is worth noting that leaves were water-saturated prior to the OP measurement and therefore, the reduction in OP under drought represents active solute accumulation per se, rather than passive water loss. During the recovery period, OP of the drought-treated plants was rapidly restored, showing values comparable to control plants after only 6 days of full irrigation.

Chl is an indicator of plant photosynthetic capacity under various environmental conditions (Fiorentini et al., 2019; Hasanuzzaman et al., 2022; Yang et al., 2023). Higher Chl under drought-stress conditions is associated with higher Chl density per unit leaf area and increased leaf thickness (Hasanuzzaman et al., 2022). Drought-treated tef exhibited increased Chl in previous studies (Girija et al., 2022; Alemu et al., 2024). In the current study, genotypes RTC-47a and RTC-290b exhibited the highest increase in Chl under drought, which was retained after recovery (**Table 1**). These two genotypes were among the three that exhibited the highest recovery in terms of CFW accumulation (**Figure 5B**), which might be related to their higher Chl.

### 4.2 Tef genotypes exhibit variation in critical drought point and recovery

The critical drought point (θ_crit_) is the soil water content below which plants fail to extract water, ultimately leading to reductions in G_sc_ and TR (Mishra et al., 2022; Appiah et al., 2023; Paul et al., 2023). Based on their stomatal plasticity in response to drought, plants are classified as either water-conserving (isohydric) or non-water-conserving (anisohydric); however, an intermediate strategy is also evident (Yi et al., 2019). Tef genotypes reflected significant variation in θ_crit_ and the reduction in TR (slope) at Cθ < θ_crit_, indicating diversity in their responsiveness to drought (**Figure 4**). RTC-275 and RTC-273b exhibited isohydric characteristics (high TR, θ_crit_ and slope), thus prioritizing water conservation over productivity, whereas RTC-359, RTC-364 and RTC-47a exhibited anisohydric characteristics (lower TR, θ_crit_ and slope), prioritizing productivity over water conservation. An intermediate strategy was displayed by RTC-290b (medium TR, θ_crit_ and slope), which might have supported its rapid recovery (**Figure 5**) and high productivity (**Table 2**).

Drought recovery refers to the plants’ physiological capacity to resume growing and producing after experiencing severe drought stress; it is considered an indicator of resilience (Fang and Xiong, 2015; Chen et al., 2016; Bongers et al., 2017; Appiah et al., 2023). Tef has the ability to maintain physiological function during drought stress and recover after water resumption, but exhibits variations in degree of recovery (Ginbot and Farrant, 2011; Kamies et al., 2017; Girija et al., 2022). In this study, tef genotypes exhibited significant variation in recovery rate (slopes) for RDT and RCFW (**Figure 5**) after 24 days of drought stress. RTC-290b displayed a high rate of recovery in both RDT and RCFW, whereas RTC-364 and RTC-47a exhibited high recovery for RCFW. Further studies are required to confirm these results and investigate recovery at later phenological stages.

### 4.3 Tef genotypes exhibits variation in productivity and WUE

The impact of water availability varies across different genotypes, reflecting their ability to adapt and respond to diverse water regimes and determining their productivity and WUE (Leakey et al., 2019). Under drought, tef growth and development are reduced, dependent on stress severity, growth stage and genetic makeup (Girija et al., 2022). In the current study, tef genotypes exhibited different performances under contrasting water regimes (**Supplementary Figure S2**, **Supplementary Tables S2**, **S3**). Drought-treated genotypes exhibited a slower accumulation of biomass (**Figure 2**) and a remarkable (~60%) reduction in their final dry weight compared to controls (**Table 2**). In our previous field experiments, tef productivity was reduced by 20–39% under drought stress (Alemu et al., 2024), similar to other studies (Admas and Belay, 2011; Abraha et al., 2017, 2018). In the field, tef develops a deep root system (Degu et al., 2008), which slows down the development of drought stress relative to growth in pots. The distribution of resources among various organs during drought stress serves as a drought-adaptive strategy (Yan et al., 2023). Drought-treated tef genotypes exhibited higher root-to-shoot mass ratio than the controls (**Table 2**), presumably as an avoidance mechanism (Zhou et al., 2018), by which they improve water uptake and reduce transpiration.

WUE involves physiological trade-offs and sensitivity to genotype-by-environment-by-management interactions (Leakey et al., 2019). Plants exhibit variations in G_sc_ and E in response to water availability, which in turn alter biomass accumulation and WUE (Sun, 2023). Being a ratio between photosynthesis and TR (or alternatively, biomass and water use), high WUE can be obtained by either increased productivity or reduced water use. Therefore, in breeding for drought resistance, it is important to combine high WUE with high productivity (Merchuk and Saranga, 2013). In the current study, WUE showed significant differences between treatments and genotypes (**Table 2**). In this respect, it is highly important that RTC-290b demonstrated the highest WUE and productivity (TDW) consistently across different water availabilities (**Table 2**).

### 4.4 Association between tef performances under greenhouse and field conditions

**Figure 6:**
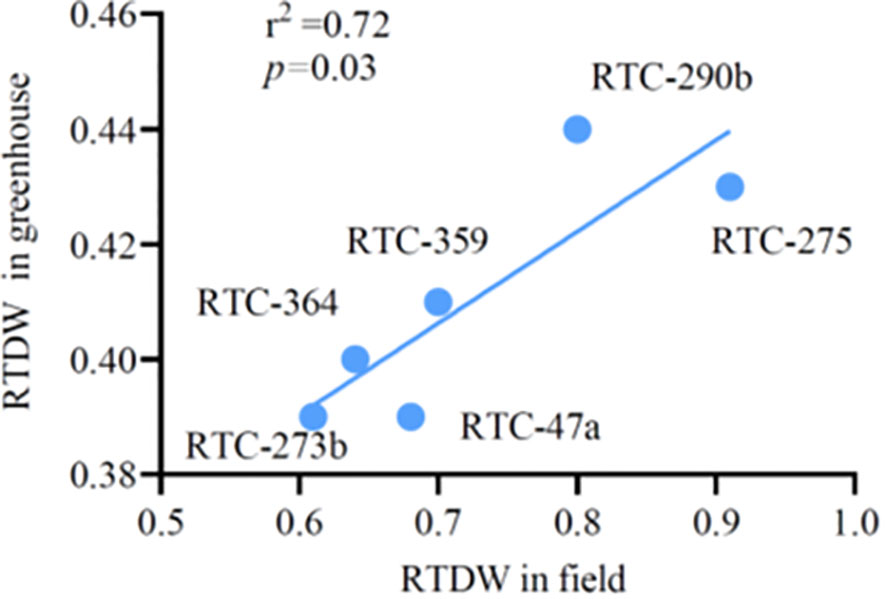
The association between relative total dry weight (RTDW) of six tef genotypes under greenhouse and field conditions.

High-throughput greenhouse-based phenotyping systems, such as the one used in the current study, can potentially accelerate functional plant phenotyping and the development of drought-resilient crop genotypes. However, it is important to confirm that the findings obtained from such systems are relevant to field conditions and determine what truly works under the relevant environments (Khaipho-Burch et al., 2023). Correlation analysis revealed a significant association (r² = 0.72, *p* = 0.03) (**Figure 6**) between RTDW values in the current pot experiment and those recorded for the same genotypes in our previous field experiment. Two genotypes (RTC-275 and RTC-290b) exhibited the highest productivity while other genotypes exhibited intermediate or low values across both experiments. It is worth noting that correlation between TDW and SDW under drought in the current study vs. TDW under drought in the field fell somewhat above the common significance threshold (p < = ~0.09). Additional studies are required to confirm these association with a wider set of genotypes and to fine-tune the greenhouse experimental procedures to better mimic plant responses in the field.

## 5 Conclusion

In the face of a rapidly growing population and changing climate, the development of more productive and stress-resilient crops has become critical (Dhankher and Foyer, 2018). Plant physiological phenotyping plays a pivotal role in understanding the intricate plant responses to environmental stress. Tef genotypes that exhibited high DT under well-watered conditions, and low DT under drought stress (e.g., RTC-275 and RTC-273b) could be advantageous where available water is ample. On the other hand, genotypes showing medium DT under well-watered conditions and high DT under drought (e.g., RTC-290b) represents better capacity to extract soil water and maintain assimilation rate, hence expected to achieve high yields under water-deficit conditions. Genotype RTC-290b, exhibited the highest DT under drought, fast recovery, an intermediate water-saving strategy and high productivity and WUE under both environments, could therefore be considered an ideotype for multiple environments. In summary, this study provided for the first time an insight into the dynamic physiological responses of tef to drought stress and revealed the variation between genotypes in drought-adaptive strategies, which may facilitate breeding of drought resilient tef cultivars.

## Data availability statement

The original contributions presented in the study are included in the article/**Supplementary Material**. Further inquiries can be directed to the corresponding author.

## Author contributions

MA: Conceptualization, Data curation, Formal analysis, Investigation, Methodology, Writing – original draft, Writing – review & editing. VB: Investigation, Writing – review & editing. IS: Investigation, Methodology, Writing – review & editing. DB: Investigation, Methodology, Writing – review & editing. YS: Conceptualization, Formal analysis, Funding acquisition, Investigation, Methodology, Project administration, Supervision, Validation, Writing – review & editing.
