# Supplementary material for: Dynamic physiological response of tef to contrasting water availabilities
Source: Front Plant Sci. 2024 Jul 9;15:1406173. doi: 10.3389/fpls.2024.1406173 (PMC11264344; doi:10.3389/fpls.2024.1406173)
Supplement: Supplementary file 1 [file DataSheet_1.docx]

**Supporting Figures**

**Dynamic Physiological Response of Tef to Contrasting Water Availabilities**

Muluken Demelie Alemu^1,2^, Vered Barak^1^, Itamar Shenhar^1^, Dor Batat^1^, Yehoshua Saranga^1^*

^1^The Robert H. Smith Faculty of Agriculture, Food and Environment, The Hebrew University of Jerusalem, Israel

^2^Ethiopian Institute of Agricultural Research, Ethiopia

* Corresponding author

E-mail: [shuki.saranga@mail.huji.ac.il](mailto:shuki.saranga@mail.huji.ac.il)

Total pages: 3

Total number of figures: 2

**
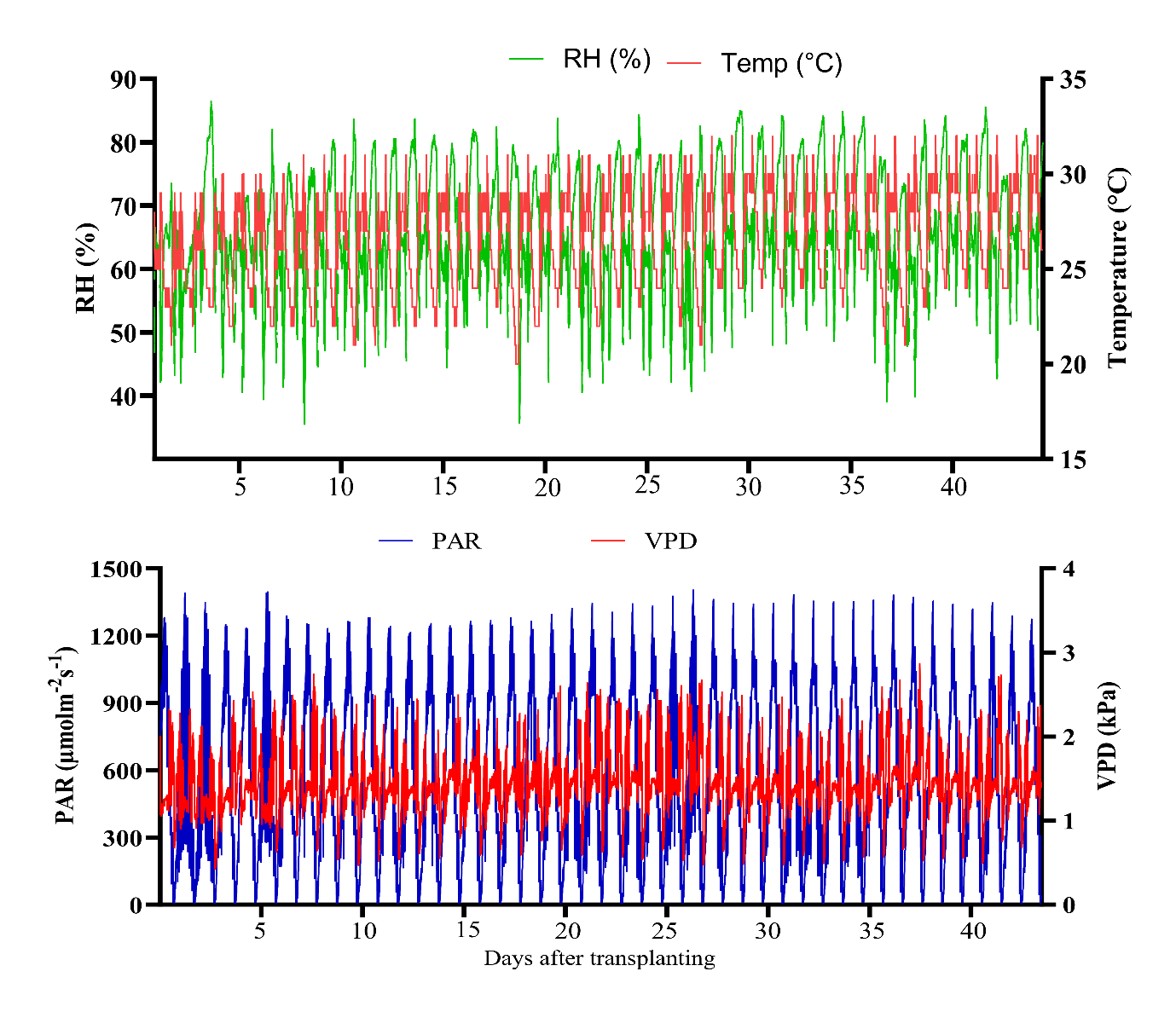
**

**FIGURE S1** Atmospheric conditions throughout the experimental season. (A) Daily temperature (red line) and relative humidity (RH, green line); (B) Daily vapor-pressure deficit (VPD, red line) and photosynthetically active radiation (PAR, blue line).


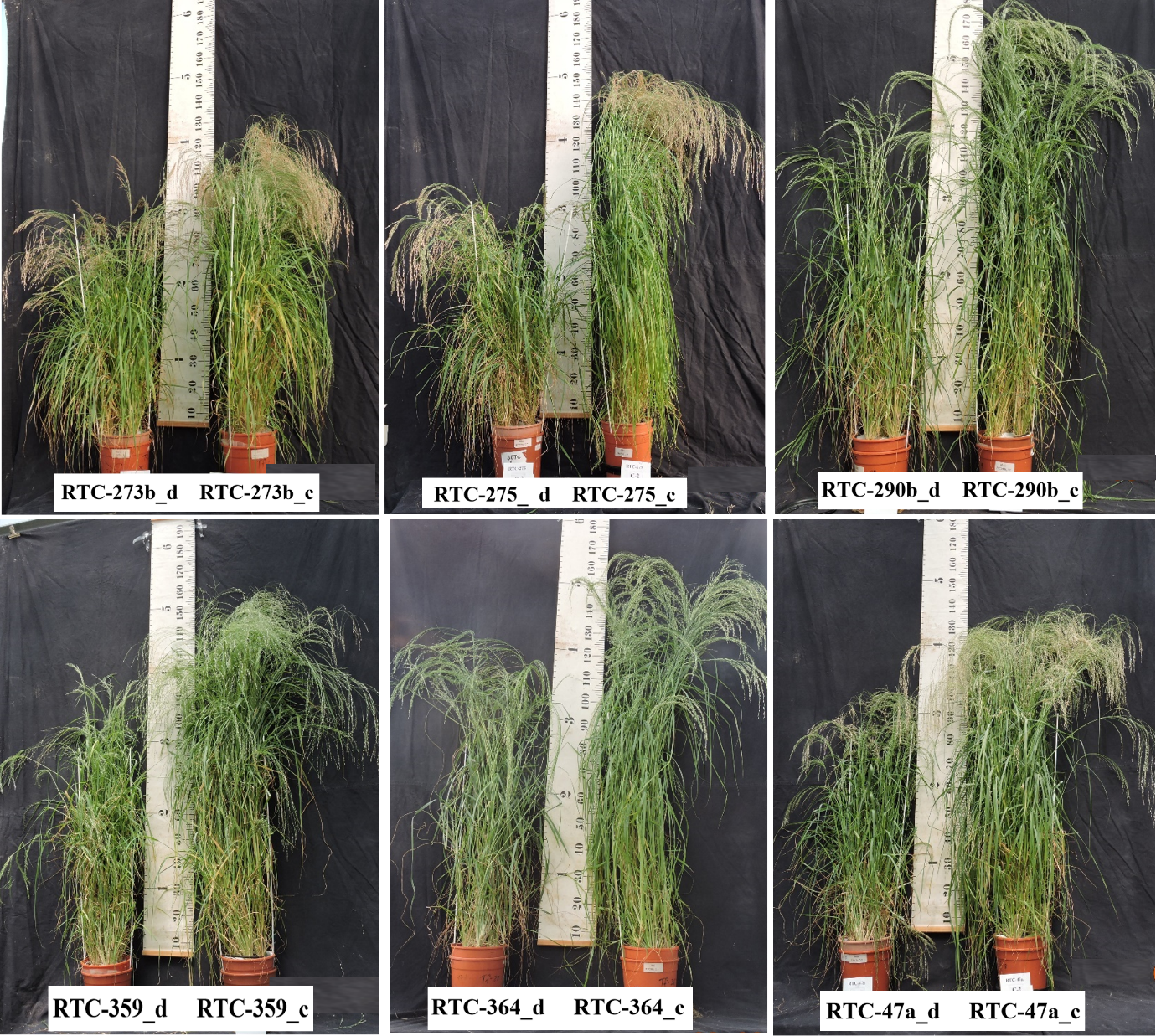


**FIGURE** **S2** Representative plants of six tef genotypes grown under drought (d) and control (c) treatment at the end of the experimental period (day 43).
